# The role of disconnection in explaining disability in multiple sclerosis

**DOI:** 10.1186/s41747-022-00277-x

**Published:** 2022-06-08

**Authors:** Caterina Lapucci, Simona Schiavi, Alessio Signori, Elvira Sbragia, Giulia Bommarito, Maria Cellerino, Antonio Uccelli, Matilde Inglese, Luca Roccatagliata, Matteo Pardini

**Affiliations:** 1 Department of Neuroscience, Rehabilitation, Ophthalmology, Genetics, Maternal and Child Health (DiNOGMI), University of Genoa, Genoa, Italy; 2 IRRCS Ospedale Policlinico San Martino, Largo P. Daneo, 3, 16132 Genoa, Italy; 3 Department of Clinical Neurosciences, Division of Neurology, Geneva University Hospitals and Faculty of Medicine, University of Geneva, Geneva, Switzerland; 4 Department of Health Sciences (DISSAL), University of Genoa, Genoa, Italy; 5 Department of Neuroradiology, IRCCS Ospedale Policlinico San Martino, Genoa, Italy

**Keywords:** Diffusion tensor imaging, Disability evaluation, Multiple sclerosis, Magnetic resonance imaging, White matter

## Abstract

**Background:**

In multiple sclerosis, the correlation between white matter lesion volumes (LV) and expanded disability status scale (EDSS) is at best moderate, leading to the “clinico-radiological paradox”, influenced by many factors, including the lack of information on the spatial localisation of each lesion on synthetic metrics such as LV. We used a probabilistic approach to provide the volume of WM tracts that may be disconnected by lesions and to evaluate its correlation with EDSS.

**Methods:**

Forty-five patients (aged 37.4 ± 6.8 years, mean ± standard deviation; 30 females; 29 relapsing-remitting, 16 progressive) underwent 3-T magnetic resonance imaging. Both LV and the volume of the tracts crossing the lesioned regions (disconnectome volume, DV) were calculated using BCBtoolkit and correlated with EDSS.

**Results:**

T1-weighted LV and DV significantly correlated with EDSS (*p* ≤ 0.006 *r* ≥ 0.413) as it was for T2-weighted LV and T2-weighted DV (*p* ≤ 0.004 *r* ≥ 0.430), but only T1-weighetd and T2-weighted DVs were EDSS significant predictors (*p* ≤ 0.001). The correlations of T1-weighted and T2-weighted LV with EDSS were significantly mediated by DV, while no effect of LV on the EDSS-DV correlation was observed.

**Conclusion:**

The volume of disconnected WM bundles mediates the LV-EDSS correlation, representing the lonely EDSS predictor.

## Key points

- Total white matter lesion volume correlates poorly with disability in multiple sclerosis.
- We quantified the volumes of the white matter tracts that crossed lesions.
- Lesioned tracts volume better correlated with disability than lesion volume.

## Background

In multiple sclerosis (MS) patients, the poor association between conventional magnetic resonance imaging (MRI) measures of tissue damage, such as T1-weighted or T2-weighted lesion load, and clinical disability represents a challenging issue both in research and clinical settings and it is widely known as “clinico-radiological paradox” [1]. The lack of histopathological specificity of MRI measures of lesion burden, as well as the lack of information about lesion location included in standard volumetric measures, represents two relevant contributors to this phenomenon.

As a matter of fact, previous studies confirmed that including information about damage location and networks disconnections may help to improve clinico-radiological correlations in MS [2]. The crucial role of anatomical disconnections between different brain regions has been also demonstrated by the evidence of microstructural transneuronal degeneration [3] and functional networks impairment [4] caused by lesions on distant cortical areas. However, most published studies that included lesion location in the composite measures of tissue damage were based on computationally intensive approaches, which reduce their clinical applicability.

The aim of our study was to obtain a simple measure, represented by the volume of the tracts disconnected by WM lesions, *i.e.*, the disconnectome volume (DV), and to investigate its correlation with the expanded disability status scale (EDSS).

## Methods

In this retrospective study, we enrolled 45 MS patients (aged 37.4 ± 6.8 years, mean ± standard deviation; 30 females, 29 relapsing-remitting and 16 secondary progressive; with an EDSS of 3.5 ± 2.0. mean ± standard deviation). The study received approval from the local ethics committee. Written informed consent was obtained from all the patients.

All patients underwent a 3-T MRI examination (General Electric Healthcare, Signa HDxt, Milwaukee, WI) with a scan protocol including the following sequences:

- three-dimensional T2-weighted fluid-attenuated inversion recovery (repetition time 3,200 ms, echo time 125.15 ms; voxel size 0.5 × 0.5 × 1 mm^3^)
- three-dimensional T1-weighted fast spoiled gradient-echo (repetition time 7.9 ms, echo time 2.7 ms; voxel size 1 × 1 × 1 mm^3^)

**Fig. 1:**
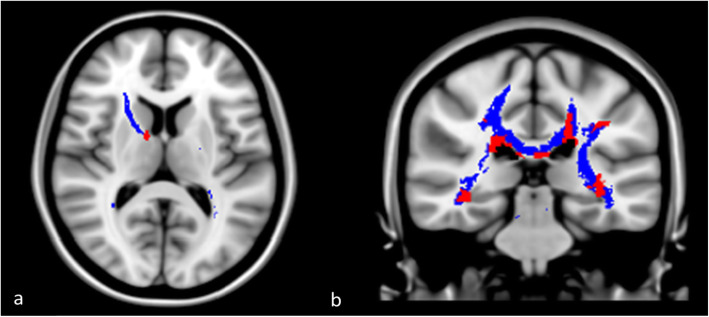
Lesion and disconnectome maps. T1-weighted hypointense lesion (*red*) and disconnectome (*blue*) maps obtained in two patients involved in the study are shown on the Montreal Neurological Institute 152 template. Axial plane (**a**): a little lesion in the genu of internal capsule (*red*) leads to a more extensive white matter bundle disconnection involving the entire anterior arm of internal capsule (*blue*). Coronal plane (**b**): multiple lesions in periventricular areas (*red*) lead to a very extensive white matter bundle disconnection (*blue*), especially in the corpus callosum

Lesion masks and volumes were obtained on both sequences by manual segmentation (Jim 7.0, Xinapse, http://www. xinapse.com, Essex, UK) after the consensus of two neurologists with 8 and 15 years of experience in MS neuroimaging (Cohen’s kappa agreement: 0.92, *p* < 0.001). T2-weighted images were linearly coregistered (NiftyReg package, CMIC, London, UK) to T1-weighted images. By using hyperintensities, lesions on T2-weighted images, and hypointense lesions on T1-weighted images as seeds, disconnectome maps were calculated with BCBtoolkit (http://toolkit.bcblab.com/) [5, 6]. (Fig. 1). This approach uses a set of diffusion-weighted imaging datasets from 35 healthy controls to obtain the tractography of a normal brain architecture [7]. Patients’ lesions in the Montreal Neurological Institute (MNI)152 space are registered to each control native space using affine and diffeomorphic deformations [8] and subsequently used as seed for the tractography. Tractographies from the lesions were thus binarised and brought to the MNI152 space using the inverse of precedent deformations. Finally, a percentage overlap map was produced by summing at each point in the MNI152 space the normalised visitation map of each healthy subject. To avoid misregistration of lesions, we visually checked all images obtained by the coregistration between patients’ lesions and healthy controls’ diffusion-weighted images. Hence, in the resulting disconnectome map, the value in each voxel takes into account the interindividual variability of tract reconstructions in controls and indicates a probability of disconnection from 0 to 1 for a given lesion [5].

**Fig. 2:**
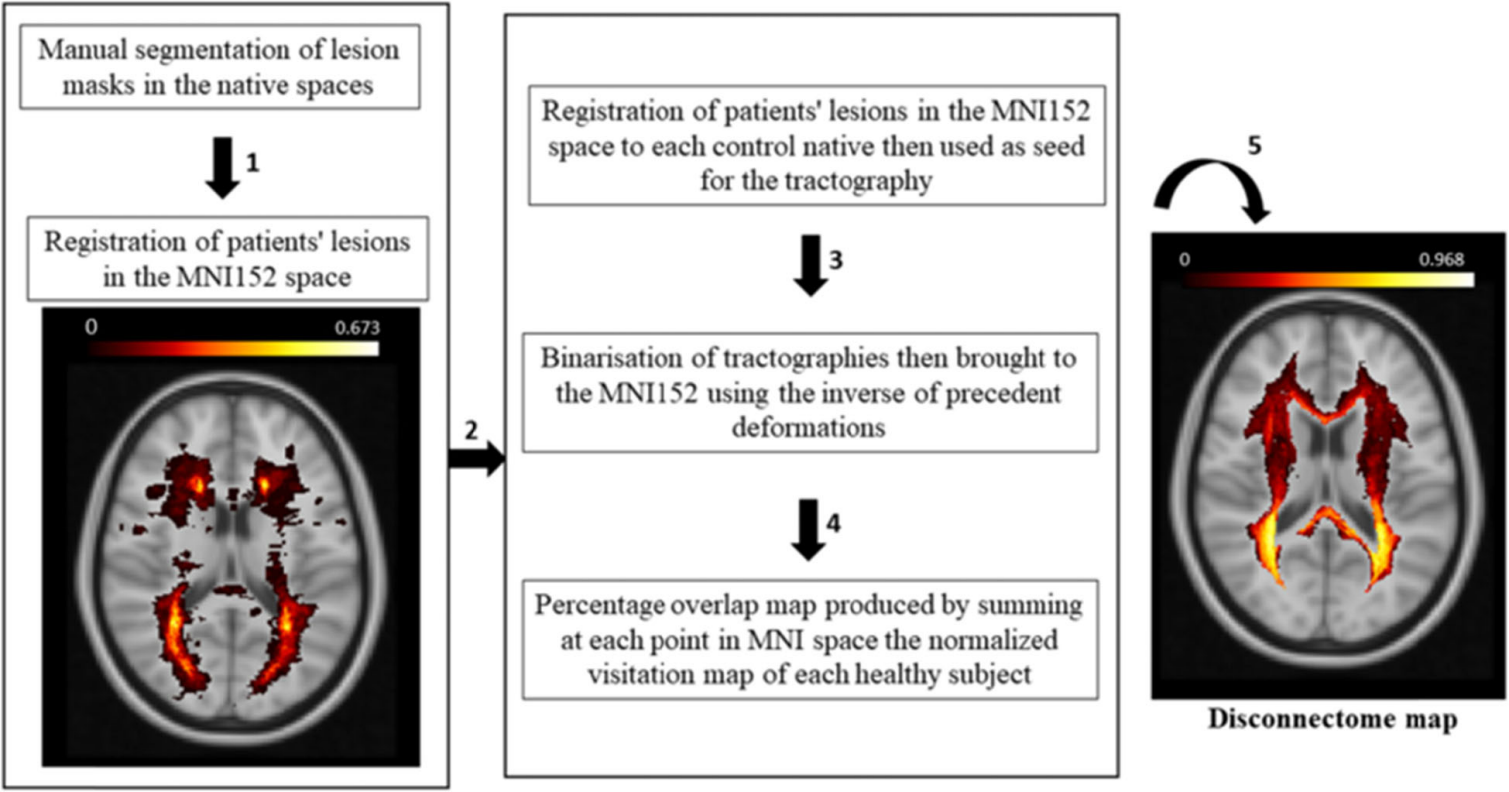
Spatial distribution of T2-weighted disconnectome maps and MRI analysis pipeline. Lesion masks were generated in native spaces and then registered in the Montreal Neurological Institute (MNI)152 space (step 1). Lesion masks in the MNI152 space were thus registered to each control native and then used as seeds for the tractography (step 2). Tractographies were binarised and then registered to the MNI152 space using the inverse of precedent deformations (step 3)**.** A percentage overlap map was produced by summing at each point in MNI152 space the normalised visitation map of each healthy subject (step 4). In the resulting disconnectome map, the value in each voxel indicates a probability of disconnection from 0 to 1 for a given lesion (step 5). The image on the left (step 1) shows the *lesion probability map*, *i.e.*, the probability for each voxel to be interested by a multiple sclerosis (MS) lesion. The image on the right (step 5) shows the *disconnectome map*, *i.e.*, the probability for each voxel to be disconnected due to MS lesions. As indicated by the colour bars, moving from red to yellow and white, the probability for a given voxel to be interested (on the left) and disconnected (on the right) by MS lesions gradually becomes more and more higher

Disconnectome volumes (DVs) were then calculated from the corresponding disconnectome maps, obtained by selecting only voxels with a probability to be disconnected of 95%. Finally, combining the DV with the standard lesion volume metrics, such as lesion volume on T1- and T2-weighted images, we assessed the strength of the correlation between the number of disconnected fibres and EDSS. MRI pipeline illustrating the processing steps with explanatory images is reported in Fig. 2.

Spearman correlations were used to assess relationships between EDSS and MRI parameters; *p* values lower than 0.05 were considered significant. Moreover, a false discovery approach was used to control for multiple comparisons. Both uncorrected and false discovery rate-corrected *p* values were reported. Stepwise bootstrapped linear regression analysis on EDSS was then performed to identify significant predictors at multivariate analyses, at first considering T1-weighted and T2-weighted measures separately. Then, as exploratory analysis, given the low sample size, a stepwise bootstrapped linear regression model of all T1-weighted and T2-weighted LV and DV data, including brain parenchymal fraction (BPF), disease duration, age, and gender as nuisance, was performed to identify significant predictors of EDSS score.

## Results

### Correlations between EDSS and T1-weighted LV or T1-weighted DV

Overall, we observed a significant correlation between EDSS and T1-weighted LV (*r* = 0.413, *p* = 0.006) as well as between EDSS and T1-weighted DV (*r* = 0.610, *p* = 0.001). In a stepwise bootstrapped linear regression model on EDSS scores, including BPF as nuisance, we found that T1-weighted DV (*p* = 0.001) but not T1-weighted LV (*p* = 0.398) was retained as a significant predictor.

### Correlations between EDSS and T2-weighted LV or T2-weighted DV

Overall, we observed a significant correlation between EDSS and T2-weighted LV (*r* = 0.430, *p* = 0.004) as well as between EDSS T2-weighted DV (*r* = 0.694, *p* = 0.001). In a step-wise bootstrapped linear regression model on EDSS scores, including BPF as nuisance, we found that T2-weighted DV (*p* < 0.001) but not T2-weighted LV (*p* = 0.698) was retained as a significant predictor, independent from BPF (Table 1).

**Table 1:** Correlations between radiological data and EDSS

Variable	Volume (mL)	Correlation with EDSS
T2-weighted LV	21.09 ± 25.04	*r* = 0.430; *p* = 0.004, *p*(FDR) = 0.006
T1-weighted LV	9.59 ± 9.43	*r* = 0.413; *p* = 0.006, *p*(FDR) = 0.008
T2-weighted DV	46.79 ± 38.2	*r* = 0.694; *p* < 0.001, *p*(FDR) = 0.002
T1-weighted DV	23.09 ± 23.53	*r* = 0.610; *p* < 0.001, *p*(FDR) = 0.002

Data are reported as mean ± standard deviation. Spearman’s correlation. *EDSS* Expanded disability status scale, *FDR* False discovery rate, *LV* Lesion volume, *DV* Disconnectome volume

### Impact of LV on EDSS and correlations with DV

After correction for LVs, the correlations of EDSS with DVs remained significant (T2-weighted DV: *r* = 0.579, *p* < 0.001; T1-weighted DV: *r* = 0.476; *p* = 0.001). Conversely, after correction for DV, the correlations of EDSS with LVs were mitigated and lost significance (T2-weighted DV: *r* = 0.530, *p* = 0.835) (Table 2). As an exploratory analysis, given the low sample size, we performed a stepwise bootstrapped linear regression model of all T1-weighted and T2-weighted LV and DV data on EDSS scores, including BPF, disease, age, and gender as nuisance. Only T2-weighted DV was retained as a significant predictor of EDSS score (*p* = 0.008). The spatial distribution of T2-weighted disconnectome maps is illustrated in Fig. 2.

**Table 2:** Partial correlations between radiological data and EDSS

Variable	Nuisance variable	Partial correlation with EDSS
T2-weighted DV	T2-weighted LV	*r* = 0.579 ; *p* < 0.001, *p*(FDR) = 0.002
T2-weighted LV	T2-weighted DV	*r = 0.15 p* = 0.530, *p*(FDR) = 0.530
T1-weighted DV	T1-weighted LV	*r* = 0.476; *p* = 0.001, *p*(FDR) = 0.002
T1-weighted LV	T1-weighted DV	*r = 0.12 p* = 0.835, *p*(FDR) = 0.84

Spearman’s correlation. *EDSS* Expanded disability status scale, *FDR* False discovery rate, *LV* Lesion volume, *DV* Disconnectome volume

## Discussion

In this study, including 45 MS patients, T1-weighted LV and DV and T2-weighted LV and DV significantly correlated with EDSS but only DVs were retained as significant predictors of EDSS scores using a bootstrapped linear regression model. Interestingly, we demonstrated that, if the impact of DV on EDSS is not affected by LV, the correlation between LV and EDSS seems to be deeply mediated by the global amount of WM tracts disconnected by MS lesions, thus strengthening our findings. Furthermore, although as a result of an exploratory analysis in which all T1-weighted and T2-weighted derived measures have been considered simultaneously, we found that, only T2-weighted DV was retained as a significant EDSS predictor, suggesting it as the possible main contributor to disconnection of WM bundles in MS.

Our findings suggest that the quantification of the whole WM fibre network involvement may help overcome the “clinico-radiological paradox”, whose basic assumption relies on the poor correlation between conventional MRI metrics of tissue damage and clinical disability [1]. Indeed, WM lesions appearance at conventional MRI represent only the “tip of the iceberg”, as a variable proportion of T2-weighted hyperintense lesions appears hypointense on T1-weighted images. In addition, T1-weighted hypointense signal itself may hide different degrees of microstructural damage [2], reflecting their histopathological heterogeneity [9]. Furthermore, lesion location and, even more, its impact on disconnection between distant brain areas, mediated by WM bundles involvement, represent further crucial issues. Indeed, there is not a linear relation between lesion burden and the amount of WM networks disruption [10], as demonstrated in a previous study in which a greater functional impairment was particularly related to the pathological involvement of WM tracts composed by a high number of fibres and located in strategical brain sites [11].

Correlative MRI clinical studies associated MS clinical aspects with a disconnection syndrome [12–14]. In a study reported by He et al. [15], network efficiency was decreased in specific regions with a relation to increased white matter lesion load.

In this study, we used the lesions masks of our patients and the tractography atlas available on BCBtoolkit [5, 6] to identify regions structurally disconnected by lesions. The impact of sample size and of age and the reliability of BCBtoolkit results to explore white matter architecture has been previously reported, demonstrating that 10 subjects are sufficient to create white matter maps sharing more than 70% of the observed variance in the general population irrespective of the age of the subjects [6].

To the best of our knowledge, a comparison between reliability of BcBToolkit and tractography from individual diffusion MRI has not yet been performed. Thus, we cannot suggest whether one approach is better than others. Nevertheless, the fact that a measure of disconnection may be obtained also without acquiring diffusion images from patients, may be accounted among BcbToolkit positives, and supports its possible applicability in multicentre studies, without the need to harmonise, acquire, and process diffusion data from different MRI scans. Although the atlas-based tractography may not adequately reflect each patient’s WM anatomy, it has been demonstrated that using healthy control diffusion-weighted imaging datasets to obtain the tractography of normal brain architecture, the network density is kept constant, and the same number of connections are analysed for each subject [16]. Finally, using tractography from individual diffusion MRI, some WM bundles may not be identified due to their interruption by lesions and thus not enter into disconnection analysis. Further studies are needed to investigate whether BcBToolkit and tractography from each patient approaches have comparable accuracy.

Notably, we assessed only the brain, thus any possible WM disconnection due to lesions involving the spinal cord was not accounted for in our tract volumetric analysis. Furthermore, brain volume disconnection due to WM lesions may only partially explain the clinico-radiological paradox in MS. Indeed, both WM microstructural integrity [2] and (whole brain and regional) atrophy [17] are involved in determining the EDSS score as an expression of clinical disability of MS patients. Due to the well-known heterogeneity of lesion location in MS and the relatively small sample size, we did not investigate the contribution of single WM tracts in explaining disability. Future studies are warranted to confirm and validate these findings in independent, prospective samples and to investigate whether the involvement of particular WM tracts may differently impact on EDSS global score, its subdomains, or other clinical correlates (*i.e.*, depression or cognitive performance). This analysis might be helpful to identify crucial sites for explaining the multiple facets of disability accrual in MS.

In conclusion, DV may give a significant contribution if added to a complex model that includes the traditional MRI measures of macro- and microstructural tissue damage and atrophy in MS patients.

## Data Availability

The datasets used during the current study are available from the corresponding author on reasonable request.

## Abbreviations

BPF: Brain parenchymal fraction
DV: Disconnection volume
EDSS: Expanded disability status scale
LV: Lesion volume
MNI: Montreal Neurological Institute
MRI: Magnetic resonance imaging
MS: Multiple sclerosis
WM: White matter
